# Impact of Dehydroepiandrosterone Sulfate on Newborn Leukocyte Telomere Length

**DOI:** 10.1038/srep42160

**Published:** 2017-02-10

**Authors:** Han Liu, Guangdi Zhou, Qian Chen, Fengxiu Ouyang, Julian Little, Jun Zhang, Dan Chen

**Affiliations:** 1 Ministry of Education and Shanghai Key Laboratory of Children’s Environmental Health, Xin Hua Hospital Affiliated to Shanghai Jiao Tong University School of Medicine, Shanghai, China; 2 School of Epidemiology, Public Health and Preventive Medicine, University of Ottawa, Ottawa, Canada.

## Abstract

The newborn setting of leukocyte telomere length (LTL) likely has important implications for telomere dynamics over the lifespan. However, its determinants are poorly understood. Hormones play an important role during pregnancy and delivery. We hypothesized that exposure to hormones may impact the fetal telomere biology system. To test this hypothesis, cortisol, estradiol, dehydroepiandrosterone sulfate (DHEAS) and reactive oxygen species (ROS) were measured in cord blood of 821 newborns from a prospective study. After accounting for the effects of potential determinants of newborn LTL, a 10-fold increase in DHEAS concentration was associated with a 0.021 increase in T/S ratio of newborn LTL (95% confidence interval: 0.009–0.034, *P* = 0.0008). For newborns who fell in the lowest quartile of DHEAS level, the mean newborn LTL was estimated to be approximately 2.0% shorter than the newborns in the highest DHEAS concentration quartile (*P* = 0.0014). However, no association was found between newborn LTL and cortisol or estradiol. As expected, newborns with higher ROS level (ROS > 260 mol/L) had lower LTL compared to that with lower ROS level (ROS ≤ 260 mol/L) (*P* = 0.007). There was also an inverse relationship between DHEAS and ROS (*P* < 1×10^−4^). Our findings suggest that exposure to DHEAS may exert a “programming” effect on the newborn telomere biology system.

Telomeres are DNA protein complexes at the ends of chromosomes, consisting of tandem TTAGGG repeats ranging from a few to 15 kilobases in length and a number of telomere binding proteins^1^. They play a central role in maintaining the integrity and stability of the genome and cell. The incomplete replication of chromosomal ends during cell division results in loss of a small fraction of telomeric DNA and makes telomere length a marker of biological age of cells, tissues/organs, and probably of the human body^2^. Telomeric DNA is synthesized by the enzyme telomerase, a ribonucleoprotein that maintains telomere function via compensating the loss of telomeric DNA. Telomerase activity is tightly regulated as, with the exception of germline cells, stem cells, and lymphocytes, most mature cells express very little telomerase^3^. In past few years, it has become well established that the telomere plays a pervasive role in regeneration of cells and tissues, physiological function, and aging^4^,^5^,^6^. Telomere shortness has been associated with common chronic diseases such as cardiovascular disease, hypertension, atherosclerosis, heart failure, and type 2 diabetes^7^, and with earlier mortality^8^.

The initial setting of leukocyte telomere length (LTL) in the newborn infant likely represents a critically important aspect of an individual’s telomere biology system. For any given individual at any age, LTL depends on, first, the initial (newborn) setting of LTL, and second, the magnitude of telomere attrition from birth onwards^9^. Therefore, a reduction in the newborn LTL could confer greater susceptibility in later life for chronic common diseases, highlighting the importance of understanding factors that determine an individual’s newborn LTL. Growing evidence suggests that conditions during early development (i.e., embryonic and fetal periods of life) interact with the genome of an individual. This interaction, in turn, influences individual’s propensity for developing one or more of the common diseases^9^. Consistent with the concept of fetal or developmental programming, newborn LTL may be plastic during development and receptive to the influence of intrauterine conditions^10^. Studies have suggested that adverse or suboptimal conditions in intrauterine life, such as preterm pre-labor rupture of the membrane^11^, maternal psychological stress^12^ and intrauterine tobacco exposure^13^ are associated with shorter offspring LTL, thereby supporting the concept that the setting of newborn LTL, may, in part be programmed in utero.

Hormones play an important role during pregnancy and delivery. We hypothesized that exposure to hormones during pregnancy and delivery may impact the fetal telomere biology system in utero, and this effect may already be apparent at birth. Hence, we investigated whether hormones (cortisol, estradiol, dehydroepiandrosterone sulfate [DHEAS]) affect newborn LTL in cord blood in the present study. We also measured the concentration of reactive oxygen species (ROS) in cord blood of all the newborns.

## Results

**Figure 1:**
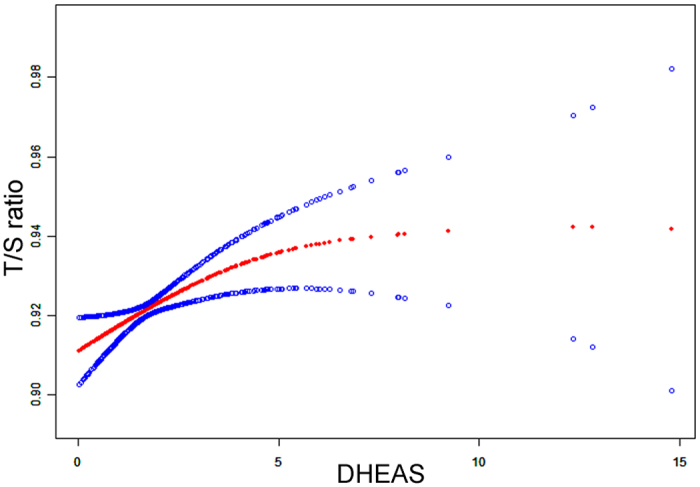
Association between DHEAS concentrations in cord blood and LTL. Spline smoothing was performed using GAM (generalized additive model) to explore the association between DHEAS concentration and newborn leukocyte telomere length after adjusting for maternal pre-pregnancy BMI, maternal and paternal ages, mode of delivery, infant sex, birth weight, gestational age at birth (days) and exposure to antepartum obstetric complications. The red points represent the fitting spline. The blue points represent the 95% confidence intervals. The unit of DHEAS concentration is μg/ml.

**Figure 2:**
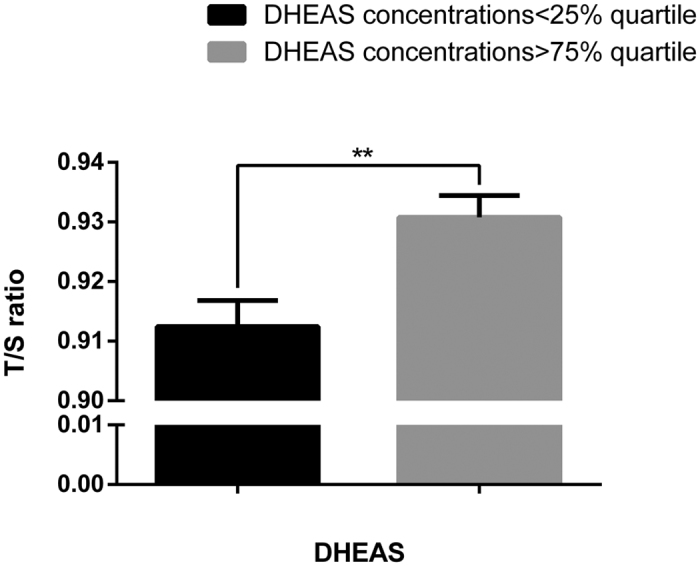
Difference of LTL in infants in the bottom vs. top quartile of DHEAS concentrations. Mean newborn leukocyte telomere length (T/S ratio, ±standard error of the mean (SEM)) for newborns who fall in the lowest quartile of cord blood DHEAS concentration vs. newborns in the highest quartile of DHEAS concentration (*P* = 0.0014). The unit of DHEAS concentration is μg/ml.

**Figure 3:**
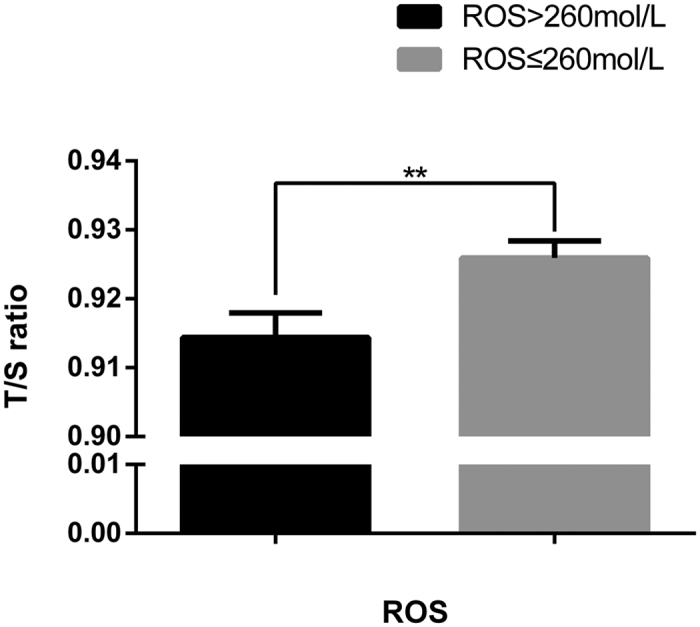
Difference of LTL in infants in the ROS > 260 mol/L vs. ROS ≤ 260 mol/L groups. Mean newborn leukocyte telomere length (T/S ratio, ±standard error of the mean (SEM)) for newborns whose ROS concentration in cord blood >260 mol/L (n = 284) vs. newborns whose ROS concentration in cord blood ≤260 mol/L (n = 537) (*P* = 0.007).

**Figure 4:**
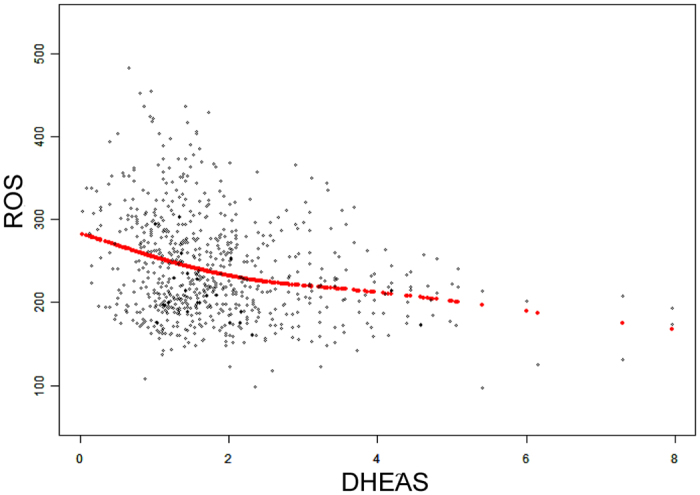
Association between DHEAS and ROS concentrations in cord blood. Scatterplot depicting the association between cord blood DHEAS concentration and cord blood ROS concentration (r = −0.205; *P* < 1 × 10^−4^). The red points comprise the smoothing spline obtained by GAM (generalized additive model) after adjusting for maternal pre-pregnancy BMI, maternal and paternal ages, mode of delivery, infant sex, birth weight, gestational age at birth (days) and exposure to antepartum obstetric complications. The blue points represent the 95% confidence intervals. The units of DHEAS concentration and ROS concentration are μg/ml and mol/L respectively.

Parental and newborn characteristics are shown in Table 1. Simple correlation analysis showed that newborn LTL was inversely proportional to gestational age at birth (days). Girls tend to have longer telomere length than boys, consistent with the finding in adults^14^. After accounting for the effects of maternal pre-pregnancy BMI, maternal and paternal ages, mode of delivery, infant sex, birth weight, gestational age at birth (days) and exposure to antepartum obstetric complications, there was a significant, independent effect of log-transformed DHEAS concentration on newborn LTL (β = 0.021; *P* = 0.0008) (see Table 2) analyzed by multiple linear regression model. Specifically, a 10-fold increase in DHEAS concentration was associated with a 0.021 increase in T/S ratio of newborn LTL (95%CI: 0.009–0.034). A smoothing spline curve, obtained by the generalized additive model and adjusted for potential confounders, shows a linear relationship between DHEAS concentration and newborn LTL (see Fig. 1). For newborns who fell in the lowest quartile of DHEAS level, the mean LTL was estimated to be approximately 2.0% shorter than the newborns in the highest DHEAS quartile (0.912 ± 0.0044 vs. 0.931 ± 0.0037 [mean ± standard error]; *P* = 0.0014) (Fig. 2). However, no association was found between newborn LTL and cortisol or estradiol. As expected, newborns with higher ROS level (ROS > 260 mol/L) had lower LTL compared to that with lower ROS (ROS ≤ 260 mol/L) (0.914 ± 0.0036 vs.0.926 ± 0.0025 [mean ± standard error]; *P* = 0.007) (Fig. 3). Furthermore, DHEAS concentration was inversely associated with ROS level (r = −0.205; *P* < 1 × 10^–4^). A scatterplot with a smoothing spline curve present the linear relationship between DHEAS and ROS concentrations in Fig. 4.

## Discussion

This study represents, to the best of our knowledge, the first report in humans to suggest that the effects of DHEAS on cellular aging may already be evident at the time of birth. This effect persists after adjusting for a number of other potential determinants of newborn LTL. The magnitude of the effect translates to an approximately 2.0% difference of LTL in infants in the top vs. bottom quartile of DHEAS concentration. Telomere length has long been considered as a biomarker of cumulative oxidative stress. Consistent with previous findings^15^, we found LTL to be inversely associated with ROS level.

Telomeres are vulnerable to oxidative damage by ROS and oxidative stress impairs the function of telomerase and other mechanisms that repair telomeres^15^. Telomere sequence which is enriched for the GGG triplet is highly sensitive to damage by ROS, with resulting 8-oxodG formation. The amount of 8-oxodG formation in DNA fragment containing telomere sequence [5′-CGC(TTAGGG)(7)CGC-3′] is approximately five times more than that in nontelomere sequence. Furthermore, ROS, especially hydroxyl radicals, can introduce single strand DNA breaks, which are less amenable to repair in telomeric than genomic DNA^16^. NO plus O^2−^ can efficiently cause base alteration at the 5′ site of 5′-GGG-3′ in the telomere sequence. Site-specific DNA damage at the GGG sequence by oxidative stress may play an important role in increasing the rate of telomere shortening with aging^17^.

There is biological plausibility for a positive association between DHEAS concentration and newborn LTL. DHEAS was inversely associated with ROS, consistent with a previous finding that DHEAS has an antioxidant effect^18^. DHEAS may protect newborn LTL through suppressing ROS. DHEAS is endogenous hormone secreted by the adrenal cortex in response to adrenocorticotrophin^19^,^20^. It circulates in the peripheral blood at relatively high concentrations, being the most abundant circulating adrenal steroid hormone in humans. Its role is still poorly understood. It likely operates in part by transformation into androgens and estradiols^21^, and probably also directly as neuroactive steroid^22^. DHEAS has long been postulated to be multifunctional hormone with antiaging, anti-inflammatory, immunomodulatory, antiatherogenic, anticancer and neurotropic effects^19^,^20^,^21^,^23^,^24^. DHEAS has been found to exert an anti-inflammatory effect in such a way that it inhibits proinflammatory cytokine-stimulated, NF-kB-mediated transcription, at least partly through its antioxidant properties^18^. Both cross-sectional and longitudinal studies have shown that in adults, serum level of DHEAS steadily declines with age^25^,^26^ and remains higher in men than in women^27^. The decline of DHEAS concentration with aging has led to the suggestion that a relative deficiency of this steroid may be causally related to the development of chronic diseases generally associated with aging, including insulin resistance^28^, obesity^19^, cardiovascular disease^20^, cancer^21^, reductions of the immune defense^22^, depression and a general deterioration in the sensation of well-being^23^. Furthermore, a lower DHEAS concentration has been found to be significantly associated with an increased mortality 2 and 4 years after DHEAS measurement in men^24^. In cord blood, DHEAS concentrations are more stable than cortisol^29^, the concentrations of which change substantially within tens of minutes. Rapidly changing concentrations are expected to increase random error, which could in part explain that there was a correlation with DHEAS but not with cortisol.

Several limitations need to be addressed in this present study. First, the initial (newborn) setting of LTL and the rate of LTL attrition are 2 key determinants of subsequent LTL. Adult LTL is in turn a determinant of aging, disease risks and mortality, it would therefore be relevant to assess the clinical significance of the subsequent LTL-related health outcomes in future longitudinal studies. Second, it is important to address the potential interactions between DHEAS and other conditions such as biophysical, medical, nutritional, and behavioral factors that could influence the setting of newborn LTL. Third, the positive association between DHEAS and telomere length may be due to confounding, i.e., an unknown factor has caused both. Further studies are warranted to rule out this possibility. Finally, we used six concentrations of the reference DNA to calculate the intra- and inter-assay coefficient of variations (CVs) instead of several different samples as reported by others^30^, which might be responsible for the low CVs observed in this study. In future work, we will use the “Shanghai Birth Cohort Study” samples to further validate the precision of our experiments.

In summary, our finding provides the first preliminary evidence in human beings that DHEAS may exert a “programming” effect on the newborn telomere biology system. Given the antioxidant effect of DHEAS, DHEAS may protect newborn LTL through suppressing ROS. Future studies are warranted to verify this speculation. Our finding adds to the evidence that age-related complex, common diseases may have their foundations very early in life.

## Methods

### Study participants and procedures

The study sample was a sub-sample of pregnant women and newborns from a larger cohort-the Shanghai Allergy Cohort which was conducted at Xinhua Hospital and International Peace Maternity and Child Health Hospital in Shanghai between 2012 and 2013. Eligible participants included permanent residents of Shanghai who had a singleton pregnancy and came to deliver in these two hospitals. Informed consent was obtained from all subjects. At enrollment, women completed a structured interview to provide information on sociodemographic characteristics and maternal behaviors while pregnant. At birth, umbilical cord blood and medical history was collected by trained nurses. At 6 months, 12 months and 24 months post-delivery, these mothers completed questionnaires on the health status of their children.

This sub-sample comprised 821 subjects in whom measures of newborn hormone concentrations and cord blood DNA were available. Comparisons of the parental and newborn characteristics between the current study population (n = 821) and the larger cohort (n = 1245) are presented in the Supplementary Table S1. No significant differences in paternal, maternal or newborn characteristics or obstetric risk conditions were found, indicating that the current study sample was representative of the larger cohort. Ethics approval was obtained by the Ethics Committees of both Xinhua Hospital affiliated to Shanghai Jiao Tong University School of Medicine and the International Maternal and Children Health Hospital. The methods were carried out in accordance with the relevant guidelines, including any relevant details.

### DNA extraction and telomere length measurement

Genomic DNA was isolated from 200 μL buffer coat of the umbilical cord blood collected in BD EDTA tubes (DNeasy Blood and Tissue Kit; Qiagen). The extracted DNA was quantified using the Epoch Multi-Volume Spectrophotometer System (Biotek) and stored at −80 °C until the time of assay. An established and validated quantitative polymerase chain reaction (qPCR) technique was used to measure the telomere length. Relative telomere length for each sample was calculated as the telomere repeat copy number[T/S]ratio using β-globin as the reference gene^31^. The definition to T/S ratio is telomere repeat copy number to single copy gene copy number. This strategy was to measure the difference between the experimental DNA sample and the reference DNA sample in its ratio of telomere repeat copy number to single copy gene copy number, so all the experimental samples were compared to the same reference DNA sample. T/S = 1 if the unknown DNA was identical to the reference DNA in its ratio of telomere repeat copy number to single copy gene copy number. Quantitative PCR was carried out in duplicate for each sample on an ABI StepOnePlus real-time PCR system (Applied Biosystems) with 20 ng DNA as a template. Each 20 μL PCR reaction contained 10 μl QuantiNova SYBR Green master mix (2×) (Qiagen) and primers for telomere length measurement: 700 nm Tel-F 5′-CGGTTTGTTTGGGTTTGGGTTTGGGTTTGGGTTTGGGTT-3′; 700 nM Tel-R, 5′-GGCTTGCCTTACCCTTACCCTTACCCTTACCCTTACCCT-3′ or primers for determinations of the single copy gene β-globin: 700 nM β-globin-F: 5′-CTTCTGACACAACTGTGTTCACTAGC-3′; 700 nM β-globin-R: 5′-CACCAACTTCATCCACGTTCACC-3′^32^. The thermal cycling profile for both telomere and single copy gene started with a 95 °C incubation for 2 minutes, followed by 40 cycles of 5-seconds denaturation at 95 °C and 1-minute annealing/extension at 60 °C. All the DNA samples were diluted to the same concentration (5 ng/μL) before the LTL measurement. Serially diluted DNA standards ranging from 0.625 to 20 ng/μL (2-fold dilution; six data points) were used to generate standard curves respectively for telomere and β-globin assays on each 96-well plate and all standard curves had a good linearity (R^2^ > 0.99). The intra- and inter-assay coefficient of variations (CVs) were 1.1% and 2.3% respectively for telomere length measurement.

### Determinations of serum ROS, DHEAS, cortisol and estradiol levels in umbilical cord blood

Total serum ROS in cord blood was measured by the use of the OxiSelect *in vitro* ROS/RNS assay kit (Cell Biolabs, San Diego, CA, USA) according to the manufacturer’s instructions. Serum DHEAS, cortisol and estradiol levels in cord blood were determined using a DHEA-S ELISA kit (LDN GmbH & Co, KG, Nordhorn, Germany), a cortisol ELISA kit (LDN GmbH & Co, KG, Nordhorn, Germany) and a estradiol ELISA kit (LDN GmbH & Co, KG, Nordhorn, Germany), respectively, according to the manufacturer’s protocol. Absorbance was measured on a spectrophotometer (Molecular Devices, Sunnyvale, CA). The intensity of the colour formed was inversely proportional to the concentration of DHEAS, cortisol or estradiol in the samples. A set of standards was used to plot a standard curve from which the amount of detected hormone concentration in samples could be directly read. Each sample was tested in triplicate.

### Statistical analyses

The Kolmogorov–Smirnov normality test was performed to examine the distribution of the continuous variables. Newborn LTL and ROS concentration were normally distributed. Hormone concentrations (DHEAS, cortisol and estradiol) tested in the serum of cord blood were log-transformed to an approximately symmetric distribution because of a skewed distribution. A univariate analysis was conducted to examine the associations of newborn LTL with different groups of related variables (Table 1) by Student’s t tests or one-way ANOVA where appropriate. Relationship among cord blood ROS, log-transformed DHEAS, cortisol, estradiol levels and LTL were analyzed by Pearson correlation analysis. Then, multiple linear regression was employed to analyse the association between log-transformed hormone concentrations and newborn LTL adjusted for maternal pre-pregnancy BMI, maternal and paternal ages, mode of delivery, infant sex, birth weight, gestational age at birth (days) and antepartum obstetric risk. Antepartum obstetric risk was defined as the presence of the following major medical complications, ie, Gestational Diabetes Mellitus, Intrauterine Growth Retardation, pregnancy-induced hypertension, preeclampsia, vaginal bleeding, placenta abruption, or infection and was coded as a ternary variable (−1 = unknown, 0 = absent, 1 = present) respectively for each complication before entering into the regression model. Risk conditions and newborn birth outcomes were obtained from the participants’ medical records. Adjustment covariates were selected a priori based on a review of the published literature on the determinants of the newborn telomere biology or based on their association with child or adult LTL^33^,^34^. These included maternal pre-pregnancy BMI, maternal and paternal ages, mode of delivery, infant sex, birth weight, gestational age at birth (days) and exposure to antepartum obstetric complications. Unstandardized regression coefficient (β) estimated the magnitude of the independent effect of that predictor on newborn LTL. Student’s t test was applied to compare the newborn LTL between those in the uppermost and lowest quartiles of DHEAS concentration, or between the ROS > 260 mol/L and the ROS ≤ 260 mol/L. All reported probability values were two-tailed and the criterion for significance was set at *P* = 0.05. Statistical analysis was performed with SAS software, version 9.2. Histograms in Figs 2 and 3 were obtained by the use of GraphPad Prism 5 software (PrismSoftwareSolutions, Inc., MN, USA). Empower(R) software (www.empowerstats.com, X&Ysolutions, Inc., Boston, MA, USA) provided the module for the plotting of Figs 1 and 4.

## Additional Information

**How to cite this article**: Liu, H. *et al*. Impact of Dehydroepiandrosterone Sulfate on Newborn Leukocyte Telomere Length. *Sci. Rep.* **7**, 42160; doi: 10.1038/srep42160 (2017).

**Publisher's note:** Springer Nature remains neutral with regard to jurisdictional claims in published maps and institutional affiliations.

## Supplementary Material

Supplementary Information

## Figures and Tables

**Table 1:** Parental and newborn characteristics (n = 821).

	Mean ± SD	N or %	T/S ratio (Mean ± SD)	*P* value
Parental characteristics				
Maternal age (years)	29.21 ± 3.51			0.483
<30		57.49%	0.923 ± 0.057	
≥30		42.51%	0.920 ± 0.060	
Paternal age (years)	31.53 ± 4.44			0.659
<31		48.11%	0.921 ± 0.058	
≥31		51.89%	0.923 ± 0.058	
Maternal prepregnancy BMI	21.34 ± 3.23			0.856
<18.5		15.96%	0.922 ± 0.057	
≥18.5 and<24		67.24%	0.923 ± 0.058	
≥24 and<28		12.78%	0.918 ± 0.058	
≥28		4.02%	0.920 ± 0.064	
Maternal education				0.266
High school or lower		15.10%	0.926 ± 0.062	
College		76.49%	0.922 ± 0.059	
Postgraduate or higher		8.41%	0.912 ± 0.046	
Paternal education				0.689
High school or lower		13.40%	0.918 ± 0.061	
College		74.91%	0.923 ± 0.058	
Postgraduate or higher		11.69%	0.922 ± 0.055	
Presence of obstetric risk condition				
Gestational Diabetes Mellitus				0.774
Yes		91	0.918 ± 0.059	
No		718	0.922 ± 0.054	
Unknown		12	0.922 ± 0.028	
Pregnancy-induced hypertension				
Yes		43	0.923 ± 0.059	0.769
No		764	0.922 ± 0.058	
Unknown		14	0.933 ± 0.049	
Preeclampsia				0.927
Yes		22	0.926 ± 0.058	
No		783	0.922 ± 0.059	
Unknown		16	0.919 ± 0.027	
Intrauterine Growth Retardation				0.563
Yes		6	0.935 ± 0.046	
No		802	0.922 ± 0.058	
Unknown		13	0.907 ± 0.050	
Vaginal bleeding				0.205
Yes		223	0.927 ± 0.056	
No		592	0.920 ± 0.059	
Unknown		6	0.898 ± 0.037	
Placenta abruption				0.27
Yes		1	0.827	
No		814	0.922 ± 0.058	
Unknown		6	0.924 ± 0.039	
Group B streptococcus (GBS) infection			0.935	
Yes		1	0.935	
No		810	0.922 ± 0.058	
Unknown		10	0.916 ± 0.040	
Urinary Mycoplasma infection				0.102
Yes		123	0.924 ± 0.064	
No		325	0.927 ± 0.062	
Unknown		373	0.917 ± 0.053	
Syphilis, gonorrhea or HIV				0.579
Yes		2	0.964 ± 0.004	
No		808	0.922 ± 0.058	
Unknown		11	0.918 ± 0.038	
Newborn characteristics				
Infant sex				0.006*
Boy		51.64%	0.917 ± 0.058	
Girl		48.36%	0.928 ± 0.058	
Birth weight (g)	3395 ± 466			0.259
<2500		2.80%	0.935 ± 0.061	
≥2500 and ≤4000		88.79%	0.922 ± 0.057	
>4000		8.40%	0.913 ± 0.064	
Gestational age (days)	274 ± 9			
<259		3.78%	0.942 ± 0.060	
≥ 259 and <280		70.03%	0.923 ± 0.058	
≥280		26.19%	0.916 ± 0.059	
Mode of delivery				0.638
Caesarean section		76.74%	0.921 ± 0.059	
Vaginal delivery		23.26%	0.924 ± 0.056	

Missing data in some variables. Groups difference of T/S ratio of every variable were compared using Student’s t tests or one-way ANOVA where appropriate.

**Table 2:** Model estimates from regressing newborn LTL (T/S ratio) on log-transformed DHEAS, Cortisol and Estradiol.

Hormone	Median (25th to 75th%)	β (95% CI)	P value
DHEAS (μg/ml)	1.61 (1.16 to 2.40)	0.021 (0.009 to 0.034)	0.0008***
Cortisol (μg/dl)	42.40 (28.76 to 63.49)	0.010 (−0.008 to 0.028)	0.269
Estradiol (pg/ml)	7957 (6279 to 10476)	0.022 (−0.001 to 0.045)	0.062

Due to the skewed distribution of DHEAS (μg/ml), Cortisol (μg/dl) and Estradiol (pg/ml), the exposure was log-transformed to an approximately symmetric distribution before entering into the regression model adjusted for maternal pre-pregnancy BMI, maternal and paternal ages, mode of delivery, infant sex, birth weight, gestational age at birth (days) and exposure to antepartum obstetric complications.
