# Supplementary material for: Impact of Dehydroepiandrosterone Sulfate on Newborn Leukocyte Telomere Length
Source: Sci Rep. 2017 Feb 10;7:42160. doi: 10.1038/srep42160 (PMC5301476; doi:10.1038/srep42160)
Supplement: Supplementary Information [file srep42160-s1.pdf]

Impact of Dehydroepiandrosterone Sulfate on Newborn Leukocyte Telomere Length

Liu Han[1], Guangdi Zhou[1], Qian Chen[1],Fengxiu Ouyang[1], Julian Little[2], Jun Zhang[1,*], Dan Chen[1,*]

Supplementary Table S1

Comparison of current study sample to larger birth cohort from which present sample was drawn. Missing data in some variables. n.s.=not significant. Groups comparison used t tests or Mann-Whitney U-tests or χ^2-tests where appropriate.

	**Current study sample(n=821)**	**Larger birth cohort(n=1245)**	**Significance of group comparison**
**Parental characteristics**			
Maternal age (years)	29.21±3.51	29.29±3.46	n.s.(*P*=0.602)
Paternal age (years)	31.53±4.44	31.72±4.63	n.s.(*P*=0.348)
Maternal prepregnancy BMI	21.34±3.23	21.23±3.15	n.s.(*P*=0.386)
Maternal education			
High school or lower	15.10%(n=124)	13.36%(n=166)	n.s.(*P*=0.518)
College	76.49%(n=628)	77.70%(n=965)	
Postgraduate or higher	8.41%(n=69)	8.94%(n=111)	
Paternal education			
High school or lower	13.40%(n=110)	13.20%(n=164)	n.s.(*P*=0.911)
College	74.91%(n=615)	74.48%(n=925)	
Postgraduate or higher	11.69%(n=96)	12.32%(n=153)	
**Presence of obstetric risk condition**			
Gestational Diabetes Mellitus	11.08%(n=91)	11.65%(n=143)	n.s.(*P*=0.961)
Pregnancy-induced hypertention	5.24%(n=43)	5.31%(n=65)	n.s.(*P*=0.985)
Preemclampsia	2.68%(n=22)	2.94%(n=36)	n.s.(*P*=0.919)
Intrauterine Growth Retardation	0.73%(n=6)	0.65%(n=8)	n.s.(*P*=0.828)
Vaginal bleeding	27.16%(n=223)	26.68%(n=330)	n.s.(*P*=0.934)
Placenta abruption	0.12%(n=1)	0.16%(n=2)	n.s.(*P*=0.974)
Group B streptococcus (GBS) infection	0.12%(n=1)	0.08%(n=1)	n.s.(*P*=0.894)
Urinary Mycoplasma infection	27.46%(n=123)	27.50%(n=146)	n.s.(*P*=0.989)
Syphilis, gonorrhea or HIV	0.24%(n=2)	0.16%(n=2)	n.s.(*P*=0.758)
**Newborn characteristics**			
Infant sex			
Boy	51.64%(n=424)	50.64%(n=629)	n.s.(*P*=0.656)
Girl	48.36%(n=397)	49.36%(n=613)	
Birth weight (g)	3395±466	3407±449	n.s.(*P*=0.541)
Gestational age (days)	274.2±9.17	274.4±8.63	n.s.(*P*=0.545)
Mode of delivery			
Caesarean section	76.74%(n=630)	74.48%(n=925)	n.s.(*P*=0.244)
Vaginal delivery	23.26%(n=191)	25.52%(n=317)	
